# Functionalized Graphene Quantum Dot Interfaced Electrochemical Detection of Cardiac Troponin I: An Antibody Free Approach

**DOI:** 10.1038/s41598-019-53979-5

**Published:** 2019-11-22

**Authors:** Muthaiyan Lakshmanakumar, Noel Nesakumar, Swaminathan Sethuraman, K. S. Rajan, Uma Maheswari Krishnan, John Bosco Balaguru Rayappan

**Affiliations:** 1 Centre for Nanotechnology & Advanced Biomaterials (CeNTAB), SASTRA Deemed University, Thanjavur, 613 401 India; 2 School of Electrical & Electronics Engineering (SEEE), SASTRA Deemed University, Thanjavur, 613 401 India; 3 School of Chemical & Biotechnology (SCBT), SASTRA Deemed University, Thanjavur, 613 401 India; 4 School of Arts, Science & Humanities (SASH), SASTRA Deemed University, Thanjavur, 613 401 India; 5 Electrodics & Electrocatalysis Division, Central Electrochemical Research Institute, Karaikudi, 630 006 India

**Keywords:** Chemical modification, Biochemical assays

## Abstract

According to the World Health Organization (WHO), cardiovascular disease (CVD) is the leading cause of death in the world every year. The design and development of biosensors for the detection of CVD markers could be one of the major contributions of the scientific community to society. In this context, acetic acid functionalized graphene quantum dots (fGQDs) were used as an interface for the electrochemical detection of cardiac Troponin I (cTnI). The interaction of cTnI with fGQDs for the early diagnosis of acute myocardial infarction was investigated using cyclic voltammetry (CV) and amperometry. The carbodiimide conjugation between the N-H group of cTnI and the functionalized COOH group on GQDs enabled the detection of cTnI biomarker. The same sensing mechanism was confirmed using Fourier Transform Infrared Spectrometry (FTIR). The fGQDs modified Au electrode showed remarkable electrocatalytic oxidation of cTnI with good stability and sensitivity over a linear range of 0.17 to 3 ng mL^−1^ and a low detection limit of 0.02 ng mL^−1^. Bland-Altman plots substantiate a bias between the intra-/inter-cTnI assay and calibrated cTnI assay with 95% limits of agreement (mean difference ± 1.96 SD). The aim of this study is to describe an innovative method to detect cardiac biomarker cTnI and provide preliminary data on its diagnostic capacity. At the same time, its applicability in clinical setting will have to be validated with a significant number of samples collected from patients.

## Introduction

Acute myocardial infarction (AMI) is one of the leading causes of death in humans. The American Heart Association reported 13.5% of sudden heart-related deaths in the United States in 2018^1^. In this scenario, spontaneous and accurate diagnosis of AMI has garnered attention among research groups. The market for cardiac biomarkers is expected to reach USD 13.3 billion by 2024^2^. Although different cardiac biomarkers are available to detect AMI, such as lactate dehydrogenase (LD), myoglobin (MB), creatine kinase-myoglobin (CK-MB) and C-reactive protein (CRP), they are less specific and sensitive than Cardiac Troponin (cT)^3^. Cardiac Troponin I (cTnI) is a “Gold Standard” biomarker for the early diagnosis of AMI because of its high sensitivity and specificity^4–6^. cTnI is released directly into the blood and its concentration in healthy people is less than 0.4 ng mL^−1^, while concentrations greater than 2.0 ng mL^−1^ indicate the presence of AMI^7^. Conventional methods used to detect cTnI are surface plasmon resonance (SPR)^8^. chemiluminescence, enzyme-linked immunosorbent assay(ELISA)^9^. electrochemical impedimetric, quartz crystal microbalance^10^, colorimetric^11^, fluorescence resonance energy transfer (FRET)^12^ and radio-immunoassay (RIA)^13^. But the main disadvantages of these methods include expensive instruments, multistage processing, and long step analysis with low sensitivity.

In this context, electrochemical biosensors with a functionalized interface could be an effective solution for the detection of cTnI^14–16^. Tungsten trioxide reduced graphene oxide nano-composite matrix was functionalized with amino groups using 3-aminopropyl tri-ethoxy saline, which covalently bonded to anti-cTnI. This immune-sensor showed a sensitivity of 58.24 mA cm^−2^ in a linear detection range of 0.01–250 ng mL^−1^ and remained stable for 30 days^17^. Similarly, 2-aminobenzyl amine functionalized graphene decorated on interdigitated electrode was fabricated to achieve amine functionalized graphene (f-GN). In order to modify f-GN electrode with monoclonal anti-cTnI, Schiff’s reaction was used. A wide linear range of cTnI detection ranges from 0.01 to 1 ng mL^−1^ with a limit of detection (LOD) of 0.01 ng mL^−1^ was reported^18^. Bhatnagara *et al*. have reported amine functionalized graphene quantum dots (afGQDs) bio-conjugated with antibody anti-cTnI to detect cTnI in the blood^19^. However, these functionalized interface-based biosensors use both antigens and antibodies which have several drawbacks, such as low recovery, efficiency, loading, high antibody production costs and lower stability at maximum temperature^20^. Hence, functionalized electrodes without antibody could be a better method for detecting cTnI in terms of the cost, stability and shelf life of the sensor.

Few antibody free approaches, such as the detection of bacteria using quantum dots-based barcode assay^21^ and detection of phosphor proteins using platinum and carbon dot hybrid nanomaterials^22^ have been reported in the literature. Similarly, in the present work, we have developed an anti-cTnI free electrochemical biosensor with acetic acid functionalized graphene quantum dots interface for early diagnosis of AMI. Since the functionalized GQDs have a high surface area, π-π conjugation, surface grafting and edge plane groups such as carboxyl groups and hydroxyl groups, they exhibit associative interactions with biological moieties^23,24^. In the present work, fGQDs are used as catalytic signal transducer and carbodiimide conjugation between fGQDs and cTnI forms the basis for cTnI detection.

The theoretically elaborated mechanism of associative interactions, as well as stoichiometric reactions of amines on activated carboxylic acid by *in situ* generated salts^25^ and experimental evidence on carboxylic acid and amine interactions induced by 9-silafluorenyl dichlorides as peptide coupling mediator with minimal wastage^26,27^ served as our motivation to develop an electrochemical biosensor with acetic acid functionalized GQDs for the detection of cTnI by enabling the interaction between carboxylic and amide groups of acetic acid and cTnI, respectively.

## Results and Discussion

### Characterization of fGQDs

**Figure 1:**
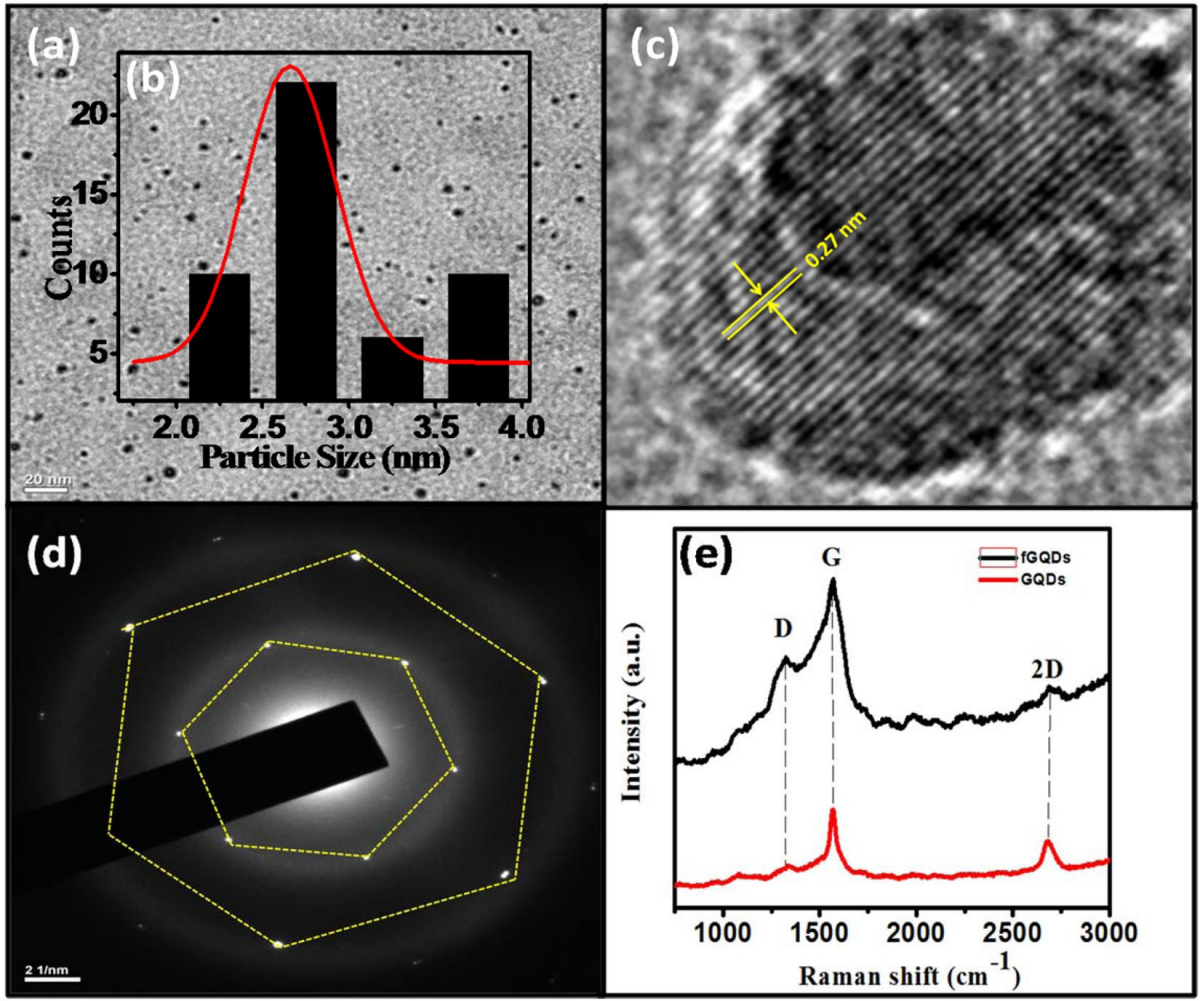
(**a**) HR-TEM image of fGQDs, (**b**) inset of fGQDs size distribution, (**c**) inter-planar spacing in fGQD, (**d**) SAED pattern of fGQD, and (**e**) Raman spectra of GQDs and fGQDs.

Figure 1(a) shows the HR-TEM image of the as-prepared fGQDs. Figure 1(b,c) shows the presence of nearly mono-disperse fGQDs of 2–4 nm and inter-planar spacing fringes of 0.27 nm. The Selected Area Electron Diffraction (SAED) pattern of as-prepared fGQDs (Fig. 1(d)) reveals a hexagonal lattice structure for the fGQD with d spacing of 0.2 nm. The crystalline nature of the sample confirms the absence of carbon quantum dots that are amorphous in nature^28,29^. The hexagonal lattice structure is typical of graphene quantum dots that have also been reported in earlier work^30,31^. Figure 1(e) shows the Raman spectra of fGQDs and GQDs for the analysis of orderness/disorderness and defects in samples. The two spectra reveal a peak at 1355 cm^−1^ (D band) due to the disorder-induced scattering confirming the imperfections in the carbon sp^2^ network induced by sp^3^ bonded atoms. The intensity of the D-band is higher in the fGQD sample than in the GQD, indicating a higher degree of disorder owing to the introduction of functional groups in the carbon network. The occurrence of a sharp peak at 1566 cm^−1^ (G band) corresponding to the E_2g_ vibration mode of sp^2^ carbon atoms in the 2D hexagonal lattice is also observed in the Raman spectra of GQD and fQGD. The I_D_/I_G_ ratio is 0.4 and 0.8 for GQDs and fGQDs, respectively, which is higher than that of pure graphite (**~**0.1)^32,33^. It is likely that the higher I_D_/I_G_ ratio for fGQD is due to increase in number of defects^34,35^. The 2D band appears around 2690 cm^−1^ in the Raman spectra of both samples. The 2D band for pristine GQD is a sharp peak with relatively higher intensity that its counterpart with a multiplet of low intensity suggesting the presence of additional layers in fGQD due to functional groups on the surface^36,37^.

### Electrochemical studies

**Figure 2:**
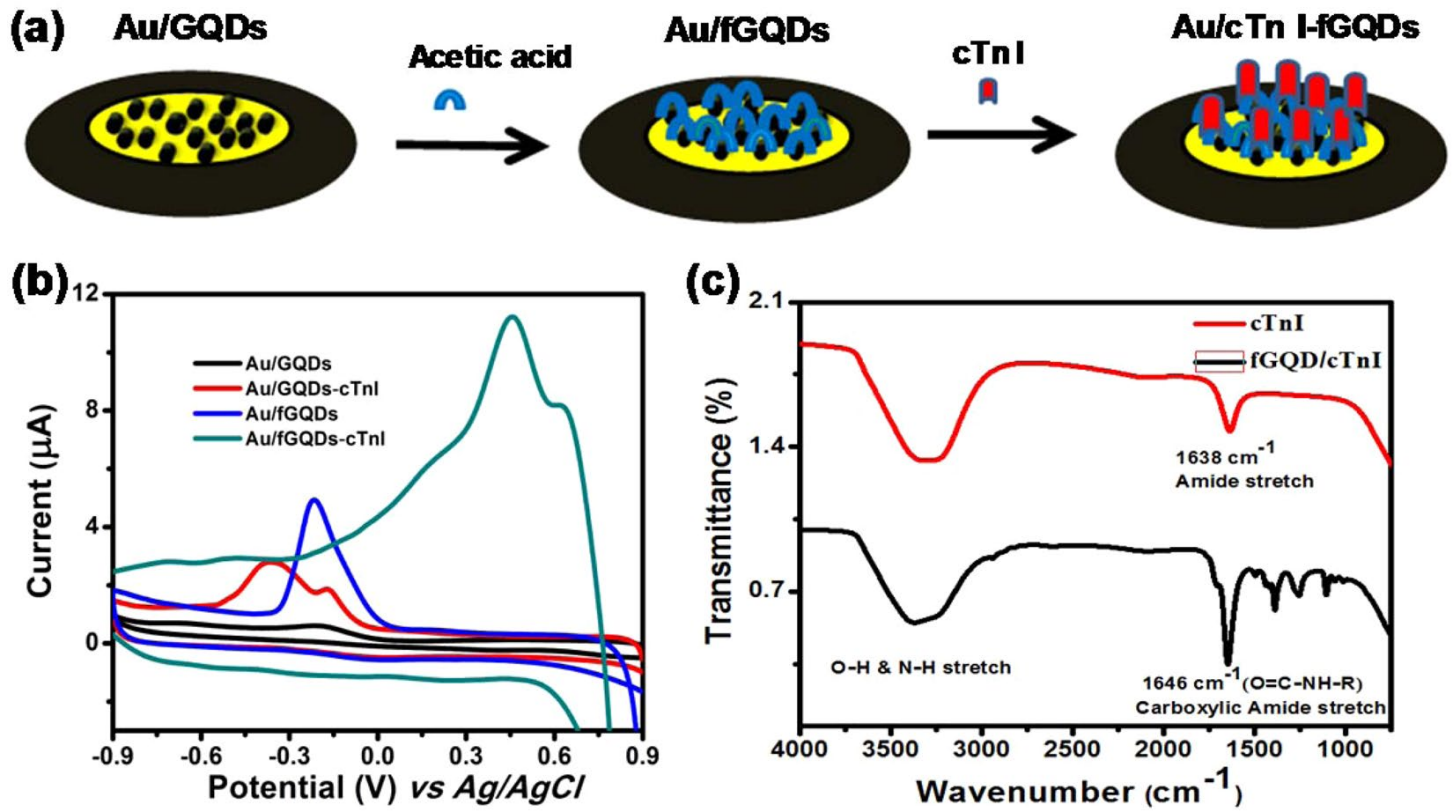
(**a**) Schematic illustration of Au electrode modification with GQDs, fGQDs & cTnI/fGQDs, (**b**) voltammogram of Au/GQDs, Au/GQDs-cTnI, Au/fGQDs and Au/fGQDs-cTnI, and (**c**) FTIR spectra of cTnI, and cTnI/fGQs.

Cyclic voltammetry (CV) studies were carried out using GQDs, fGQDs, cTnI/GQDs and cTnI/fGQDs modified Au working electrodes (Fig. 2(a)) in 0.1 M NaCl solution. No redox peak was observed for the Au/GQD electrode, whereas the Au/fGQD electrode had an anodic peak at −0.2 V, indicating the electrocatalytic property of the immobilized fGQDs. The catalytic properties of fGQDs have been well-documented in the literature^23,24^. In the presence of cTnI, twin anodic peaks at 460 and 623 mV were observed in the Au/fGQD electrode (Fig. 2(b)). The occurrence of these peaks may be explained based on the amino acid residues in the cTnI sequence and associative interactions existing between the amide functionalities of cTnI, as well as the hydrogen bonding interactions of the peptide with the aqueous electrolyte.

The Au-GQD electrode in comparison also shows a similar pattern of peaks but with lower intensity and at a lower potential. Cardiac troponin I is a 210 amino acid protein containing approximately 49% charged amino acids (glutamate, aspartate, lysine, arginine or histidine) and 11% of hydroxyl group containing amino acids (serine, thereonine, tyrosine) and, hence, it can interact effectively through hydrogen bonding interactions with the functionalized GQDs more effectively that with a GQD. As a result, greater retention of cTnI is achieved on fGQD through the covalent interactions when compared to GQD. The presence of charged amino acid residues in cTnI contributes to the higher current, whereas the positive shift in the peak potentials on fGQD can occur due to the hydrogen bonding interactions between the peptide and fGQD. The FT-IR spectrum of cTnI (Fig. 2(c)) reveals a broad band centred at 3275 cm^−1^ due to the –O-H and –N-H stretching vibrations. The broad band suggests the presence of extensive hydrogen bonding. The amide carbonyl stretch appears prominently at 1638 cm^−1^. The FTIR spectrum of cTnI incorporated fGQD shows a broad vibration band due to –N-H around 3400 cm^−1^, while the –O-H vibration appears as a shoulder around 3260 cm^−1^ ^38^. The carbonyl (C=O) stretch observed at 1646 cm^−1^ can be attributed to the amide functional group of the protein, while the carboxylic acid functionalization of the fGQD is discernible as a shoulder at 1715 cm^−1^. The schematic representation of the mechanism of carbodiimide conjugation between peptide chains and water molecules in the electrolyte is shown in Fig. S1 ^39^.

**Figure 3:**
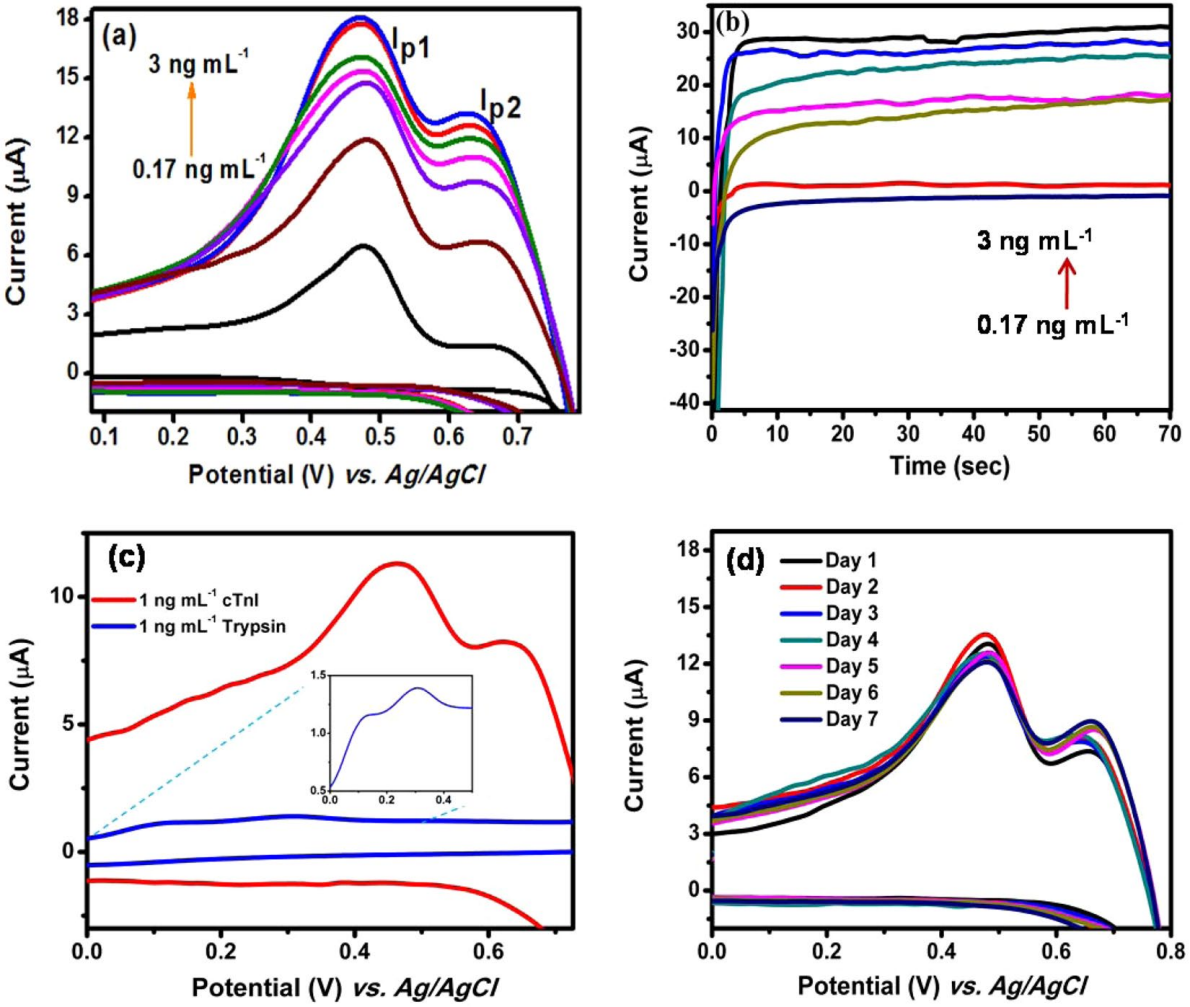
(**a**) Cyclic voltammogram of Au/fGQDs electrode for different cTnI concentrations, (**b**) amperometric current response for different cTnI concentrations at 460 mV, (**c**) cyclic voltammogram of Au/fGQDs electrode using trypsin interferent and (**d**) reproducibility (n = 3) study.

**Figure 4:**
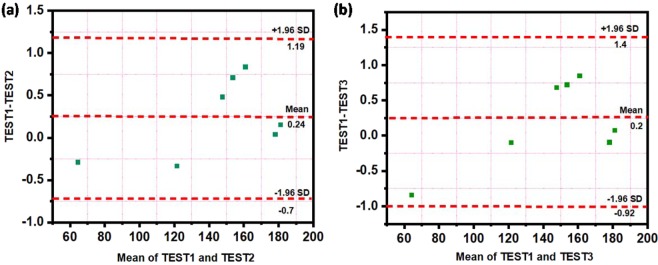
(**a**) Bland Altman plot between TEST1 (calibrated cTnI assay) & TEST2 (intra cTnI assay) and (**b**) TEST1 & TEST3 (inter cTnI assay) as a function of two corresponding tests. The middle dotted line is mean; upper and lower dotted lines are the limit of agreement.

The cyclic voltammogram of Au/fGQDs electrode for different concentrations of cTnI was recorded at an optimized scan rate of 0.01 Vs^−1^. The oxidation peak currents (I_p1_ and I_p2_) were found to increase with increasing cTnI concentration from 0.17 to 3.0 ng mL^−1^ (Fig. 3(a)). The low detection limit (LOD) was found to be 0.02 ng mL^−1^ for I_p_ at 460 mV, which is attributed to the hydrogen bonding between the amide and fGQD and the additional shoulder anodic peak I_p2_ corresponds to C=O–H-O hydrogen bonding between amide-water at 623 mV. Johansson *et al*. have theoretically reported that “amide-amide hydrogen bonding is stronger than amide-water bonding”^40^. Both the calibration plots (I_p1_ and I_p2_) exhibited a linear relationship between various concentrations of cTnI. The observed anodic oxidation peak current confirmed the surface-controlled process (R^2^ = 0.99) (Fig. S2(a,b)) and amperometry response was observed for different cTnI concentrations (0.17 to 3.0 ng mL^−1^) at 460 mV. A response time of 10 s was noted and the current attained a steady state, as shown in Fig. 3(b). To verify the anti-interference ability of the sensor, the CV response was recorded in the presence of another protein trypsin and was shown in Fig. 3(c). Twin peaks similar to those observed for cTnI was observed for trypsin but at different potentials of 140 and 300 mV and with reduced magnitude. Trypsin contains 240 amino acid residues of which about 18% are charged (glutamate, aspartate, lysine, arginine or histidine) and 16% are hydroxyl containing amino acids. This suggests that the amount of trypsin retained on the surface of fGQD will be lower owing to lesser non-convalent interactions. In addition, the lower number of charges on the trypsin reduces the magnitude of current recorded at the Au/fGQD surface. This study clearly confirmed that cTnI can be detected without antibody at the specific potential of 460 mV using Au/fGQDs electrode. In order to confirm reproducibility, sensing performance of the fabricated Au/fGQDs electrodes was studied and a relative standard deviation (RSD) of 3.1% was obtained as shown in Fig. 3(d). In order to assess the bias between the mean differences and to estimate an agreement interval, within which 95% of the differences between the intra-/inter-cTnI assay and calibrated cTnI assay falls, Bland and Altman analysis is performed. As can be seen from Fig. 4(a,b), the maximum allowed differences between TEST1 and TEST2, TEST3 were within the limit of agreement, suggesting that the proposed cTnI assay is in agreement with intra- and inter-cTnI assay standards.

To the best our knowledge, there are no articles on antibody-free electrochemical detection of cTnI. However, there is a unique report that uses the surface plasmon resonance technique for the detection of cTnT, also a biomarker for Acute Myocardial Infarction (AMI) in which Palladino *et al*.^41^, employed the epitope imprinted surface towards antibody free point of care testing for AMI. They reported a LOD of 15.4 nM, which is almost 10^3^ times higher than the LOD obtained by fGQDs modified Au electrode (0.02 ng mL^−1^). In this context, the proposed electrochemical detection method can pave the ways for affordable real-time testing with an effective, antibody free tool for inexpensive point-of-care devices to detect the cardiac biomarkers assays.

## Conclusion

The diagnostic capacity of an antibody-free fGQDs-interfaced electrochemical biosensor for the detection of cTnI has been reported. The hydrogen bonding interactions mediated by the carboxylic group in fGQDs played an important role in the detection of cTnI. The developed Au/fGQDs sensor displayed sensitivity over a linear range of 0.17–3 ng mL^−1^ and a low detection limit of 0.02 ng mL^−1^. Bland and Altman analysis was carried out for the proposed cTnI assay shown 95% agreement between the intra-/inter-cTnI assay and calibrated cTnI assay. Even though this antibody-free biosensor was sufficiently sensitive and stable in detecting cTnI, an extensive validation on a pre-determined number of patients are required before its use can be considered in a clinical setting.

## Experimental

### Materials

Human heart cTnI protein reagent, trypsin and nafion were procured from Sigma-Aldrich, India. Graphite was purchased from Alfa Aesar, India. Sodium chloride, sodium nitrate, sulphuric acid, hydrazine monohydrate, potassium permanganate, hydrogen peroxide, dimethylformamide and acetic acid were procured from Merck, India.

### Apparatus

The structural morphology of fGQDs was studied using a field emission transmission electron microscope (FE-TEM)(JSM 2100 F, JEOL, Japan). The degree of orderness/disorderness and defects of fGQDs were identified using Raman spectrometer (Horiba Xplora, Japan). The interaction between immobilized fGQDs and human cTnI was evaluated using attenuated total reflectance (ATR) mode in the Fourier Transform Infrared (FT-IR) spectrometer (Alpha T, Bruker, Germany) in the spectral range of 1300–3600 cm^−1^ with a spectral resolution of 8 cm^−1^. Cyclic voltammetry and amperometry measurements were carried out using CH600C electrochemical workstation (CH Instruments Inc., USA) with CH600C software. Electrochemical experiments were conducted using three electrode systems with platinum wire as the auxiliary electrode, Ag/AgCl saturated in 0.1 M KCl as the reference electrode, and fGQD modified gold electrode (fGQD/Au) as the working electrode. All experiments were performed at ambient temperature (300 K).

### Synthesis of fGQDs

Graphene Oxide (GO) was synthesized using the modified Hummer’s method. 1 g graphite and 0.8 g sodium nitrate were added to 30 mL of sulphuric acid and continuously stirred in an ice bath for 4 h. Subsequently,4 g of potassium permanganate was slowly mixed with the solution and stirred at room temperature for 1 h. 100 mL of deionized water was added to the solution and warmed at 310 K for 1 h. The further reaction was terminated using (10 mL) 30 w/v% of hydrogen peroxide. The formation of GO was indicated by a sudden transformation of the black coloured solution into brownish colour. The prepared GO was washed several times with 5 w/v% of HCl and filtered GO was dried for 24 h^42^. For the synthesis of GQDs, 1 g of GO was added to 100 μL dimethylformamide, 1 μL hydrazine monohydrate and stirred at room temperature for 12 h. The solution was ultrasonicated for 24 h and then stirred for 1 h at 353 K. Functionalized graphene quantum dots (fGQDs) were prepared by adding 10 mg of GQDs with 20 w/v% of acetic acid, 0.5% of nafion (100 μL) and ultrasonicated for 3 h at 353 K.

### Fabrication of Au/fGQDsElectrode

The electrode kinetics was improved by removing the oxide layer and the contaminants on the surface of Au working electrode. 0.05 µm alumina powder was used to polish the electrode surface and pretreated by soaking in 1.0 M KOH and 10 w/v% of HNO_3_ for 15–20 min. Later, as-prepared fGQDs were ultrasonicated for 2 h and 3 μL of fGQDs were drop casted on the surface of the bare Au working electrode. Finally, the fabricated Au/fGQDs working electrode was dried at room temperature for 3 h.

## Supplementary information

Supplementary information
