# Supplementary material for: Functionalized Graphene Quantum Dot Interfaced Electrochemical Detection of Cardiac Troponin I: An Antibody Free Approach
Source: Sci Rep. 2019 Nov 22;9:17348. doi: 10.1038/s41598-019-53979-5 (PMC6874552; doi:10.1038/s41598-019-53979-5)
Supplement: Supplementary file 1 — Supplementary information [file 41598_2019_53979_MOESM1_ESM.docx]

**Supplementary Information:**

**Functionalized Graphene Quantum Dot Interfaced Electrochemical Detection of Cardiac Troponin I: An Antibody Free Approach**

Muthaiyan Lakshmanakumar^1,2^, Noel Nesakumar^5^,Swaminathan Sethuraman^1,3^, K.S. Rajan^1,3^, Uma Maheswari Krishnan^1,3,4^, and John Bosco Balaguru Rayappan^1,2*^

^1^Centre for Nanotechnology & Advanced Biomaterials (CeNTAB), ^2^School of Electrical & Electronics Engineering (SEEE), ^3^School of Chemical & Biotechnology (SCBT),

^4^School of Arts, Science & Humanities (SASH)

SASTRA Deemed University,Thanjavur – 613 401, India.

^5^Electrodics & Electrocatalysis Division, Central Electrochemical Research Institute,

Karaikudi–630 006, India.

The schematic representation of the carbodiimide conjugation between the peptide chains and water molecules in the electrolyte

Fig. S1. Schematic mechanism of carbodiimide conjugation.

Both calibration plots (I_p1_ and I_p2_) exhibited the linear relationship between various concentrations of cTnI and anodic oxidation peak current (R^2^=0.99).

**_
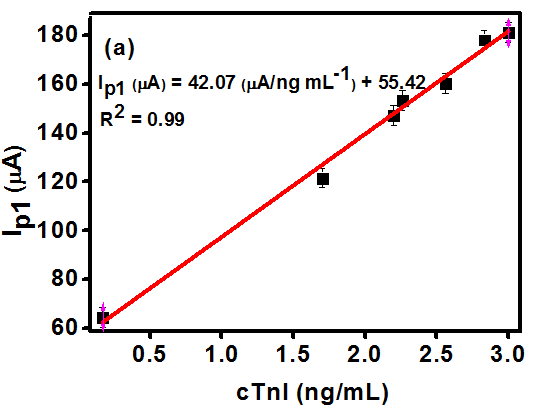
_**

Fig. S2.(a) Calibration curves of I_p1_.

**_
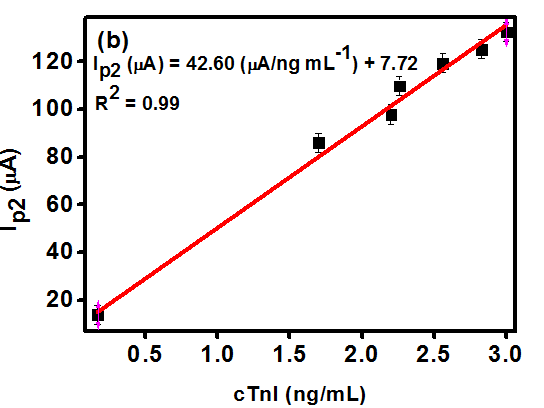
_**

Fig. S2.(b) Calibration curves of I_p2_.
